# A game changer for bipolar disorder diagnosis using RNA editing-based biomarkers

**DOI:** 10.1038/s41398-022-01938-6

**Published:** 2022-05-04

**Authors:** Nicolas Salvetat, Francisco Jesus Checa-Robles, Vipul Patel, Christopher Cayzac, Benjamin Dubuc, Fabrice Chimienti, Jean-Daniel Abraham, Pierrick Dupré, Diana Vetter, Sandie Méreuze, Jean-Philippe Lang, David J. Kupfer, Philippe Courtet, Dinah Weissmann

**Affiliations:** 1 ALCEDIAG/Sys2Diag, CNRS UMR 9005, Parc Euromédecine, Montpellier, France; 2 Les Toises. Center for Psychiatry and Psychotherapy, Lausanne, Switzerland; 3 Department of Psychiatry, University of Pittsburgh School of Medicine, Pittsburgh, PA USA; 4 Department of Psychiatric Emergency & Acute Care, Lapeyronie Hospital, CHU Montpellier, Montpellier, France

**Keywords:** Bipolar disorder, Diagnostic markers

## Abstract

In clinical practice, differentiating Bipolar Disorder (BD) from unipolar depression is a challenge due to the depressive symptoms, which are the core presentations of both disorders. This misdiagnosis during depressive episodes results in a delay in proper treatment and a poor management of their condition. In a first step, using A-to-I RNA editome analysis, we discovered 646 variants (366 genes) differentially edited between depressed patients and healthy volunteers in a discovery cohort of 57 participants. After using stringent criteria and biological pathway analysis, candidate biomarkers from 8 genes were singled out and tested in a validation cohort of 410 participants. Combining the selected biomarkers with a machine learning approach achieved to discriminate depressed patients (*n* = 267) versus controls (*n* = 143) with an AUC of 0.930 (CI 95% [0.879–0.982]), a sensitivity of 84.0% and a specificity of 87.1%. In a second step by selecting among the depressed patients those with unipolar depression (*n* = 160) or BD (*n* = 95), we identified a combination of 6 biomarkers which allowed a differential diagnosis of bipolar disorder with an AUC of 0.935 and high specificity (Sp = 84.6%) and sensitivity (Se = 90.9%). The association of RNA editing variants modifications with depression subtypes and the use of artificial intelligence allowed developing a new tool to identify, among depressed patients, those suffering from BD. This test will help to reduce the misdiagnosis delay of bipolar patients, leading to an earlier implementation of a proper treatment.

## Introduction

The current COVID-19 pandemic has led populations to a lockdown whose demographic and social impacts are still underevaluated. Health restrictions are already causing ravages in the field of psychiatry, with an explosion of anxiety and depression disorders [1]. Indeed, recent studies demonstrate a huge increase of depression prevalence since COVID pandemic [2, 3]. Furthermore, the spread of COVID-19 and important death rate may aggravate the risk of mental health issues and intensify current psychiatric symptoms of certain individuals who are on risk of anxiety, depression, stress, and violence [4]. Thus, the need for a reliable and accurate differential diagnosis, allowing an adequate treatment, of these pathologies has become a crucial necessity for the coming years. Depression is one of the most common mental health disorder affecting near 10% of men and 20% of women worldwide [5], and is associated with a significant increased mortality, mostly due to suicidal behavior [6]. The Diagnostic and Statistics Manual for Mental Disorders characterizes major depressive episode using a combination of five or more different symptoms, e.g. depressed mood, anhedonia, sleep dysregulation, fatigue or indecisiveness [7, 8]. Within mood disorders, bipolar disorder (BD) is one of the most frequent and disabling ones, affecting 1% of the world’s population, characterized by episodes of mania, hypomania, and alternating or intertwining episodes of depression. In a primary care clinic, 21% of patients being treated for depression screened positive for BD and 2/3 of them reported that they have never been diagnosed bipolar [9]. Indeed, Judd and colleagues have shown that patients were manic or hypomanic less than 10% of the time and without symptoms about half of the time, meaning that they were depressed during 40% of the time [10]. As a consequence, the average interval between onset of BD symptoms and proper diagnosis is estimated to be around 7 years [11], delaying suitable treatment and care management, and increasing suicide risk [12, 13]. Various clinical interview-based instruments are available and routinely used in practice by psychiatrists to diagnose BD, including evaluation of manic symptoms by Young Mania Rating Scale (YMRS) [14], the Altman self-rating scale (ASRM) [15] or the Mood Disorder Questionnaire (MDQ) [16]. Biological markers to set the boundaries between the different subtypes of depression are lacking and a major research goal is to identify reliable and clinically useful biomarkers to differentiate BD from unipolar depression [17, 18].

Recent studies have shown an association between depression and RNA alterations by epitranscriptomic mechanisms [19], including RNA methylation [20], microRNAs [21], and RNA editing [22–24]. One of the most studied processes occurring at the RNA level is the Adenosine (A)-to-inosine (I) conversion, mediated by ADARs (Adenosine deaminase acting on RNA) which bind to double stranded RNA (dsRNA) stem loop and modify A to I by deamination [25]. Inosine is interpreted as guanosine by the cellular machinery due to their similar chemical characteristics. RNA editing can thus induce single amino acid substitutions in coding regions, leading to new start or stop codons or modifying splicing sites. Furthermore, it can also affect RNA stability by modifying the Untranslated Regions UTRs [26, 27] as well as the formation of different microRNAs isoforms [28, 29]. Significant difference in RNA editing has already been reported in neurological or immune diseases, among other pathologies [30]. In the Central Nervous System (CNS), permeability of ion channels and responses to excitatory neurotransmitters have been found to be altered by RNA editing [31]. Recently, we have identified in the anterior cingulate cortex (Brodmann Area 24) of depressed suicide decedents modifications in 5-HTR2c (5-hydroxytryptamine receptor 2c) [32] and PDE8A (PhosphoDiEsterase 8 A) [33] mRNA editing. Interestingly, phosphodiesterases, a key modulator of signal transduction downstream 5-HTR2c, is involved in inflammatory cell activation, memory and cognition [34]. More recently, we demonstrated the diagnostic value of PDE8A mRNA editing in depression, in the blood from HCV patients treated with interferon-α [35], and in the blood from depressed patients and suicide attempters compared to age-matched and sex-matched healthy controls [36].

In the current study, we focused on transcriptome-wide RNA editing modifications detected by RNA sequencing (RNA-Seq) to identify new genes with differential A-to-I RNA editing in blood samples from depressed patients as compared to healthy controls (*n* = 57, discovery cohort). The diagnostic potential of this panel of edited RNA was validated on 410 samples (validation cohort) from either healthy controls (*n* = 143) or depressed patients (*n* = 267) by ultra-deep Next Generation Sequencing (NGS). The same approach was applied by dichotomizing the depressed patients in unipolar (*n* = 160) or bipolar (*n* = 95) patients to identify specific RNA editing sites or RNA editing isoforms (referred as to biomarkers) on specific RNA sequences (gene targets). The diagnostic performances of their combination was evaluated via a machine learning approach to differentiate unipolar versus BD patients.

## Materials and methods

### Subjects and clinical assessment

**Fig. 1:**
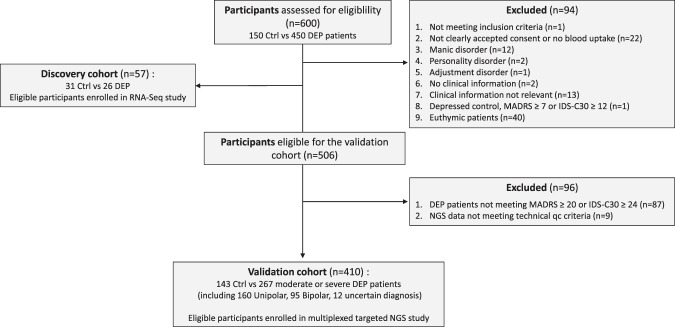
STARD flow chart diagram of participants included in the study. The diagram reports the flow of participants through the study, indicating the number of participants evaluated for the study, the number of participants excluded because they did not meet the inclusion criteria, and the number of participants included in the discovery and in the validation study.

Depressed patients (DEP) were recruited from September 2016 to January 2019 among the outpatients of the Department of Emergency Psychiatry and Post-Acute Care (CHRU of Montpellier) according to the principles of the Helsinki Declaration of 1975 and its successive updates. This study was approved by the French local Ethical Committee (CPP Sud-Méditerranée IV in Montpellier, CPP No.A01978-41) and registered under the reference identifer NCT02855918. All participants, aged between 18 and 65 years, understood and signed a written informed consent before entering the study. All patients met the MDD criteria in Diagnostic and Statistical Manual of Mental disorders IV (DSM-IV) using the Mini-International Neuropsychiatric Interview [37]. During the standardized interview, psychiatrists managed the French version of the Montgomery-Åsberg Depression Rating Scale (MADRS) [38] and the 30-item Inventory of Depressive Symptomatology, Clinician Rated (IDS-C30) [39] to score depression. Manic symptoms were evaluated by the (YMRS) [14]. Depression severity levels, i.e. low, moderate and severe were defined by 7 ≤ MADRS ≤ 19 and/or 12 ≤ IDSC-30 ≤ 23, 20 ≤ MADRS ≤ 34 and/or 24 ≤ IDSC-30 ≤ 36, MADRS ≥ 35 and/or IDSC-30 ≥ 37, respectively. Presence of BD was assessed by clinician’s expertise. Two independent cohorts were used in our study: a discovery cohort (*n* = 57), which was used for RNA-Seq experiments and biomarker discovery and a validation cohort (*n* = 410), which was used for Targeted Next Generation Sequencing, biomarker validation and algorithm settings. All the patients received a treatment classified into these 5 categories: anxiolytics, hypnotics and sedatives, antidepressants, antipsychotics, and antiepileptics (Table 1). Age-, race- and sex-matched control subjects were recruited from a list of volunteers from the Clinical Investigation Center (CHRU of Montpellier). The complete flow of the patients through the study is shown in Fig. 1 and their complete description in Table 1.

**Table 1:** Demographic and clinical characteristics of the study population included in the validation cohort.

	Total Sample	Controls	Depressive			
			DEP	Unipolar	Bipolar	Uncertain
Number, n	410	143	267	160	95	12
Age						
Age (min-max)	18–65	19–65	18–64	18–64	19–64	24–62
Age (mean ± SD)	39.3 ± 13.4	39.4 ± 14.1	39.3 ± 13	36.3 ± 13.4	44.3 ± 10.9	38.5 ± 11.8
* p* value (vs Ctrl)			0.95			
* p* value (vs UN)					<0.00001	
Gender						
Male (n(%))	133 (32.4)	53 (37.1)	80 (30)	50 (31.2)	26 (27.4)	4 (33.3)
Female (n(%))	277 (67.6)	90 (62.9)	187 (70)	110 (68.8)	69 (72.6)	8 (66.7)
* p* value (vs Ctrl)			0.61			
* p* value (vs UN)					0.95	
Inflammation						
CRP, mg/l (mean ± SEM)	2.8 (±0.3)	2.1 (±0.4)	3.2 (±0.5)	2.9 (±0.6)	3.6 (±0.7)	4.9 (±1.7)
* p* value (vs Ctrl)			0.053			
* p* value (vs UN)					0.44	
Clinical characteristics						
MADRS score (mean ± SEM)		0.8 (±0.1)	29.7 (±0.5)	30.7 (±0.6)	28.2 (±0.7)	29.8 (±1.6)
* p* value (*vs* Ctrl)			<0.00001			
* p* value (vs UN)					0.02	
IDSC30 score (mean ± SEM)		1.97 (±0.2)	36.6 (±0.5)	37.4 (±0.7)	35.3 (± 0.8)	34.8 (± 2.0)
* p* value (*vs* Ctrl)			<0.00001			
* p* value (vs UN)					0.050	
YMRS score (mean ± SEM)				1.4 (±0.21)	2.7 (± 0.45)	
* p* value (vs UN)					0.02	
BMI, kg/m2 (mean ± SEM)		24.6 (±0.4)	23.8 (±0.3)	23.1 (±0.4)	24.9 (±0.5)	
* p* value (*vs* Ctrl)			0.11			
* p* value (vs UN)					0.01	
Psychotropic treatments						
Anxiolytics (n (%))	181 (44.1)	0	181 (67.8)	110 (68.8)	60 (63.2)	11 (91.7)
Hypnotics/Sedatives (n (%))	43 (10.5)	0	43 (16.1)	22 (13.8)	18 (18.9)	3 (25)
Antidepressants (n (%))	174 (42.4)	0	174 (65.2)	117 (73.1)	47 (49.5)	10 (83.3)
Antipsychotics (n (%))	123 (30)	0	123 (46.1)	57 (35.6)	61 (64.2)	5 (41.7)
Antiepileptics (n (%))	59 (14.4)	0	59 (22.1)	17 (10.6)	41 (43.2)	1 (8.3)
* p* value (vs UN)					<0.00001	
Substances addiction						
Tobacco (n (%))	161 (39.3)	26 (18.2)	135 (50.6)	78 (48.8)	50 (52.6)	7 (58.3)
Alcohol (n (%))	34 (8.3)	0	34 (12.7)	24 (15)	8 (8.4)	2 (16.7)
Other Substances (n (%))	40 (9.8)	0	40 (15)	25 (15.6)	11 (11.6)	4 (33.3)
* p* value (vs Ctrl)			0.0012			
* p* value (vs UN)					0.27	

Data are the mean ± SEM. *p* values of main characteristics are obtained with the Student’s *t* test or chi-squared test. Controls: Healthy volunteers; Depressive: patients suffering from depression.

*CRP* C reactive protein, *MADRS* Montgomery-Åsberg depression rating scale, *IDS-C30* The 30 item Inventory of Depressive Symptomatology, *YMRS* Young Mania Rating Scale, *BMI* body mass index.

### RNA extraction and qualification from whole blood

Samples were retrieved in PAXgene™ blood RNA tubes, distributed randomly in different sets of extractions and isolated using MagNA Pure 96 instrument (Roche) (LifeScience), according to the manufacturer’s protocol. Total RNA concentrations and quality levels were determined with Qubit Fluorometer (Invitrogen) and LabChip (Perkin-Elmer, HT RNA Reagent Kit) instruments, respectively. Only samples with RNA integrity values>7 were taken into consideration for further analysis.

### Library preparation for RNA-sequencing in discovery cohort

For RNA-Seq library we used a TruSeq Stranded Total RNA library kit (Illumina), specifically tailored for blood samples, according to the manufacturer’s instructions. Briefly, 300 ng total RNAs were depleted in rRNA and globin mRNA (Ribo-Zero Globin), purified and fragmented into 250 bp fragments in average. First strand of cDNA was synthetized using Superscript II Reverse Transcriptase (Thermo Fisher Scientific, random primers) and the second strand cDNA using DNA Polymerase I and RNase H (UTP incorporation). DNA fragments were selectively enriched to obtain the library, then normalized, denatured (0.1 M NaOH) and sequenced (High-Output, 2 × 75 bp read length) on an Illumina NextSeq 500. Approximately 70 million reads per sample were acquired.

### Bioinformatics analysis of A-to-I RNA editome data

Paired-end reads generated by Illumina NextSeq 500 were demultiplexed using bcl2fastq (version 2.17.1.14, Illumina). Sequencing quality was performed using FastQC (version 0.11.7) and MultiQC software [40]. Identification and quantification of A-to-I editing events were performed using RNAEditor (Version 1.0) with default parameters [41]. RNAEditor accepts FASTQ files as input and implements a workflow fully automated, which comprises a mapping step with BWA [42] using the human genome version (GRCh38 release-83 [43]), followed by a step of PCR duplicates removal, a local realignment and a step of base quality score recalibration with Genome Analysis Tool Kit (GATK4) [44]. For detection of RNA editing events, Unified Genotyper of GATK4 is used followed by several steps of purifications to reduce the false positives number (known SNPs are excluded, variants in splice junctions are erased, variants in homopolymers are removed) and finally, a step of RNA editing events annotation. We used high confident editing filtering criteria for further analysis (base quality >25, mapping quality>20, mean/median coverage > 30x, min edited reads≥2, editing degree change ≥10%, remove editing with 100% editing degree and Wilcoxon’s test *p* value < 0.05). Functional annotation of edited events was then performed with ANNOVAR [45], RepeatMasker [46] (http://repeatmasker.org) and REDIportal [47]. Alu editing index (AEI) was performed according to the methodology described in Bazak and al [48]. A flowchart of our A-to-I RNA editome analysis is shown in Supplementary Fig. 1A.

### Gene-disease association analysis

The 7 identified genes have been analyzed by the DisGeNET v6.0 (http://www.disgenet.org), which integrates human gene-disease associations (GDAs) from one of the largest publicly available collections of genes and variants associated to human diseases [49, 50]. The investigated diseases belong to one or more of the following MeSH categories: mental disorders, behavior and behavior mechanisms, nervous system diseases and immune system diseases. We used “disgenet2r” R package (version 0.0.9) to analyze and visualize DisGeNET data results.

### Targeted next generation sequencing in validation cohort

Regions of interest for each gene were amplified with validated primers using Q5 Hot Start High Fidelity enzyme (New England Biolabs) according to manufacturer guidelines, on a Peqstar 96x thermocycler. PCR products were purified with magnetic beads (High Prep PCR MAGbio system, Mokascience), amplicon purity was determined with Nucleic Acid Analyzer (LabChipGx, Perkin Elmer) and then quantified (Qubit system, ThermoFisher Scientific). After indexing samples (Q5 Hot Start High fidelity PCR enzyme, Nextera XT index kit of Illumina), the library was pooled, purified (Magbio PCR cleanup system), denatured (0.1 M NaOH), spiked with PhiX Control V3 (Illumina), loaded onto a sequencing cartridge (Illumina MiSeq Reagent Kit V3 or Illumina NextSeq 500/550 Mid-Output) according to Illumina’s guidelines, and finally sequenced (1 ×150 bp read length) at standard concentrations using ultra-deep sequencing. Approximately 1 million reads per sample were acquired.

### Bioinformatics analysis of targeted sequencing data

A description of the bioinformatics pipeline used in this study has already been detailed [35] and a corresponding flowchart is shown in Supplementary Fig. 1B. The sequencing data were downloaded from the Illumina NextSeq 500 and their quality was checked using FastQC software (version 0.11.7, https://github.com/s-andrews/FastQC/). A minimal sequencing depth of 20,000 reads for each sample and each target was considered for further analysis. A pretreatment step was performed consisting of removing adapter sequences and filtering off the sequences according to length and quality score. Short reads (<100nts) and reads with an average QC < 20 were removed. To improve sequence alignment quality, flexible read trimming and filtering tools for Illumina NGS data were used (fastx_toolkit v0.0.14 and prinseq version 0.20.4). After performing pre-processing steps, an additional quality control of each cleaned fastq file was carried out prior further analysis. Alignment of the processed reads was performed using bowtie2 [51] (version 2.2.9) with end-to-end sensitive mode. The alignment was done to the reference human genome sequence GRCh38. Non-unique alignments reads, unaligned or reads containing insertion/deletion (INDEL) were removed from downstream analysis by SAMtools software [52] (version 1.7). SAMtools mpileup [53] was used for SVN calling. Finding edited position in the alignment was done by using in-house scripts in order to count the number of different nucleotides in each genomic location. For each position, the script computes the percentage of reads that have a ‘G’ [Number of ‘G’ reads/ (Number of ‘G’ reads + Number of ‘A’ reads)*100]. The genomic location ‘A’ reference with percentage in ‘G’ reads > 0.1 are automatically detected by the script and are considered as ‘A-to-I edition site’. The last stage is to compute the percentage of all possible isoforms of each transcript. By definition the relative proportion of RNA editing at a given editing ‘site’ represents the sum of editing modifications measured at this unique genomic coordinate. Conversely, an edited mRNA isoform is a unique molecule that may or may not contain multiple editing modifications on the same transcript. For example for a given transcript, the edited mRNA isoform BC contains an A-to-I modification on both site B and site C within the same transcript. We considered as biomarker a RNA editing site or isoform with a significant diagnostic value in either Ctrl vs DEP or UN vs BD comparison. A relative proportion of at least 0.1% was set as the threshold in order to be included in the analysis.

### Statistical analysis of data

All statistics and figures were computed with the “R/Bioconductor” statistical open source software [54, 55]. Biomarker values are presented as mean ± standard error of the mean (SEM). To prevent putative bias, all biomarkers were adjusted for batch effect removal and clinical covariates as age, substances addiction and psychotropic treatments using ComBat method (sva R package, version 3.33) [56]. In order to guarantee normally distributed data, each biomarker data was transformed using bestNormalize R package (version 1.4.2) [57]. A differential analysis was carried out using the most appropriate test between the Mann–Whitney rank-sum test, Student’s *t* test or Welch’s t test according to normality and sample variance distribution. A *p* value below 0.05 was considered as statistically significant. A “Target Editing Index” (TEI), resuming gene-specific editing values, was computed by linear combination of significant editing variants which maximizes AUC (Area Under the curve) ROC (Receiver Operating Characteristic) [58, 59]. In addition, all biomarkers were combined with each other to evaluate the potential increase in sensibility and specificity using random forest (RF), a machine learning approach [60]. This method requires the use of a training set used to construct the model and a test set to validate it. We have shared our data set: ~70% of the dataset are used for the learning phase and 30% are used for the testing phase. This sharing has been randomized and respects the initial proportion of the various statutes in each set. RF method combines Breiman’s “bagging” idea and the random selection of features in order to construct a collection of decision trees with controlled variance. To prevent bias of unbalanced cohort, Multiple Down-Sizing (MultDS) approach was implemented [61]. This method randomly draws samples from the majority class but includes all samples from the minority class for one tree. To generalize the model, we performed 100 learning RF trees. The final classifier was generated by summing up the votes (probabilities) for each applied sample from each tree, and normalized by the number of trees. The end resulting probabilities reflect the majority vote of the 100 RF trees. We used a grid learning approach for each individual tree, where we stated certain maximum parameter sets (ntree = 1000, nodesize = 25 and mtry = (1,100)). The subtrees are trained with a classical 10-fold cross-validation. RF results are shown on the test dataset which has never seen the algorithm. The implementation was done using the R randomForest package (version 4.6-14) and R caret package (version 6.0-84) [62].

### Functional enrichment analysis

Functional enrichment analysis of differential A-I RNA editing variant genes was performed using ontologizer (http://ontologizer.de) and reactome analysis tools (https://reactome.org/). Gene Ontology (GO, www.geneontology.org) Biological Processes and Reactome gene sets were used to determine the functions associated with our selected genes

## Results

### Characteristics of the patients

Depressed patients were classified according to clinical scores in MADRS and IDSC-30 depression scales. We observed a significant association between these scores in both discovery and validation cohorts (*p* < 0.0001, Supplementary Fig. 2A and B respectively), suggesting a correct assignment of patients included in the study and a relative homogeneity in the evaluation of depression. Patients included in the discovery cohort were matched by gender and show no statistical differences between groups in term of age or BMI (Supplementary Table 1). In validation cohort (Table 1), unipolar patients have significantly higher depression scores than bipolar patients (MADRS and IDS-30), while YMRS is significantly higher in BD group. Noteworthy, this score is very low in BD group, showing these patients are not in a manic phase in this study. The inflammation marker CRP was not statistically significant between groups, conversely to age and BMI level. About 68% of depressed patients are treated with anxiolytics, and 16% with hypnotics and sedatives. Concerning antidepressants, 65.2% of patients are treated, mainly unipolar depressed patients while 46.1% of patients received antipsychotics, mainly BD patients. To prevent a putative bias in further analyses due to differences in medications, all results were adjusted by age, sex, psychiatric treatments and substances addiction.

### Editome analysis

**Fig. 2:**
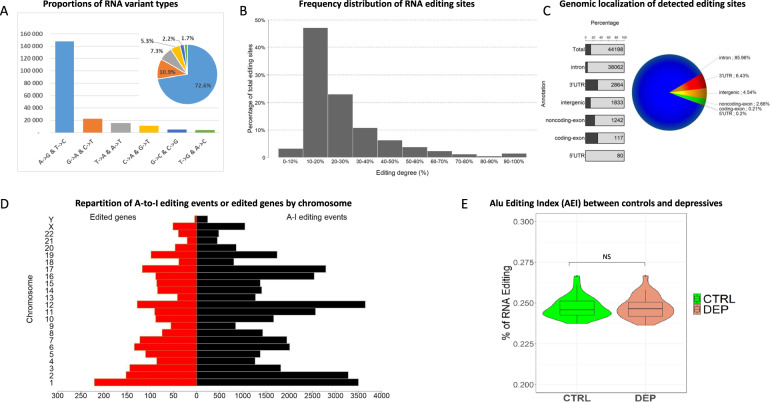
RNA editing landscape in whole blood. **A** Proportions of RNA variant types in human whole blood. A-to-G variant, indicating A-to-I editing, is disproportionately enriched. **B** The percentage of RNA editing sites across different intervals of editing degrees. **C** The proportion and numbers of A-to-I editing sites across genomic localization are shown in piechart and at the right of the gray bars. The proportion of editing sites not residing in Alu repeats is represented by black bars. **D** Repartition of A-to-I editing events or edited genes by chromosome or gene. Black (right y-axis): Repartition of A-to-I editing events by chromosome. Red (left y-axis): Number of edited genes by chromosome. **E** Distribution of Alu editing index (AEI) values in controls and depressed patients. Data are the mean ± SEM. *p* value of AEI index was calculated using the Wilcoxon rank-sum test.

Using our RNA editome pipeline, we identified 40,398 A-to-I edited positions with high degree of confidence in at least one sample. Two major variant types were identified (A-to-G and T-to-C variants), which represent 72.6% of the RNA variants (Fig. 2A). Most A-to-I editing sites presented moderate editing degree, where 10–30% editing degree accounted for the largest proportion (Fig. 2B). This RNA edition is found mainly in introns and in 3′ untranslated regions (UTRs) (Fig. 2C) as well as in Alu repeat regions and has a homogeneous repartition all over the genome (Fig. 2D). Finally, no significant difference in Alu editing index was observed between control and depressed patients (Fig. 2E).

### Biomarker identification

**Fig. 3:**
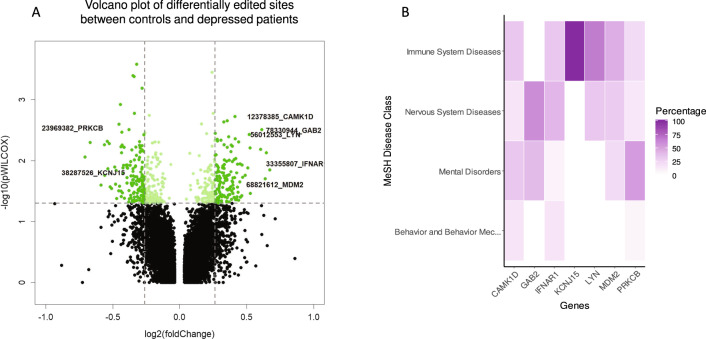
Identification of differentially A-to-I RNA edited sites between healthy controls and depressed patients using RNA-Seq data from human blood. **A** Volcano plot of differentially edited sites between healthy controls and depressed patients. The volcano plot shows the upregulated and downregulated sites differentially edited between depressed patients healthy controls. For each plot, the x-axis represents the log2(Fold Change) (FC), and the y-axis represents -log10(*p* values). Editing sites with a *p* value of < 0.05 were assigned as differentially edited and are indicated in green. Significant editing sites of selected genes (see Table 2) are labeled in black. **B**: Heatmap of MeSH class associated with the 7 genes identified by RNAseq annotated by the DisGeNET database. All identified genes have been analyzed and their relationships with mental disorders, behavior and behavior mechanisms, nervous system diseases and immune system diseases MeSH categories have been calculated with DisGeNET database. The darker the heatmap, the stronger the association.

In our study the terms “biomarker” refers to an editing site or isoform of RNA that contains one or more positions differently edited in UN vs BD comparison. Differential RNA editing analysis was carried out on a discovery cohort between patients suffering from depression (*n* = 26) and controls (*n* = 31). As shown by volcano plot analysis, we identified 646 variants differentially edited, representing 366 genes (Fig. 3A).

To further narrow down the list of biomarkers, and obtain a reduced and more significant panel, more stringent quality inclusion criteria were applied and combined: (i) Sites not located in intergenic regions, nor near known SNPs (± 100 bp); (ii) Editing sites present in at least 25% of samples; (iii) Median/mean coverage ≥30X; (iv) RNA Editing 0.8 ≥ FoldChange ≥ 1.20; (v) Significance with *p* value < 0.05; (vi) AUC ROC > 0.7. Functional enrichment analysis was then performed by hypergeometric test (FDR < 0.05). We focused on biological processes of Gene Ontology (GO) [63] and Reactome Pathways [64]. Both analyses showed strong term enrichment for multiple immune categories (Supplementary Tables 2 and 3, Supplementary Figs. 3 and 4). Gene disease association analysis (DisGeNET) confirmed the functional analysis and all genes were associated with at least one disease belonging to one of the MeSH categories tested (Fig. 3B), leading to 7 editing sites candidates, each representing one target gene (Table 2, Supplementary Fig. 5 and Supplementary Tables 4 and 5): CAMK1D (Calcium/calmodulin-dependent protein kinase type 1D); GAB2 (Growth factor receptor bound protein 2-associated protein 2); IFNAR1 (Interferon alpha/beta receptor 1); KCNJ15 (ATP-sensitive inward rectifier potassium channel 15); LYN (Tyrosine-protein kinase Lyn); MDM2 (E3 ubiquitin-protein ligase Mdm2); PRKCB (Protein kinase C beta type).

**Table 2:** Gene and RNA editing variants identified by RNA-Seq between depressed patients (DEP, *n* = 26) and controls (CTRL, *n* = 31) in the discovery cohort.

Ensembl ID	GeneName	Region	Chr	Position (GRCh38)	Coverage	Editing CTRL	Editing DEP	Fold change	Fold median	*P* value	AUC ROC
ENSG00000183049	CAMK1D	intron	10	12378385	46	17	21	1.28	1.33	2.20E–03	0.796
ENSG00000033327	GAB2	intron	11	78330944	37	17	24	1.43	1.52	3.71E–03	0.896
ENSG00000142166	IFNAR1	3UTR	21	33355807	32	17	24	1.42	1.24	1.21E–02	0.910
ENSG00000157551	KCNJ15	intron	21	38287526	85	22	18	0.83	0.75	1.85E–02	0.737
ENSG00000166501	LYN	3UTR	8	56012553	44	15	20	1.35	1.33	4.37E–03	0.774
ENSG00000135679	MDM2	intron	12	68821612	36	12	16	1.29	1.29	2.89E–02	0.797
ENSG00000166501	PRKCB	intron	16	23920896	78	16	12	0.74	0.69	3.43E–03	0.853

Shown are the gene ID, gene name, chromosome and information on the genomic localization of detected editing sites. The position (GRCh38) of editing site identified in RNA Seq study and its Coverage, Mean editing values by conditions (in percentage), Fold Change, Fold Median, *p* value and AUC ROC are indicated.

### Biomarker validation

**Fig. 4:**
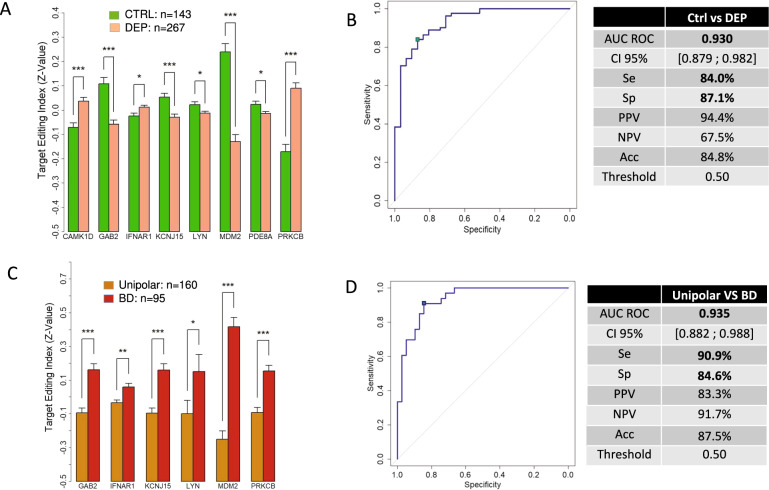
Diagnostic performance of the tests. Target Editing Index (TEI) were calculated by combining all significant RNA editing variants with *p* value ≤ 0.05 for each target gene. ROC curve plotted are the probabilities for the correct response of the tested sets using case specific trained random forest models. The results of the 100 trained random forest by MultDS were combined by majority voting for test dataset. RF results are shown on the test dataset which has never seen the algorithm. The implementation was done using the randomForest and caret R package. **A** TEI for depressed patients (DEP; *n* = 267) and healthy controls (CTRL; *n* = 143); (**B**) ROC curve and diagnostic performance of Random Forest model for DEP vs CTRL classification; (**C**) TEI for unipolar (*n* = 160) and bipolar disorder (BD; *n* = 95); (**D**) ROC curve and diagnostic performance of Random Forest model for unipolar vs BD classification.

In addition to the 7 identified targets, we selected PDE8A on the basis of our previous results [33, 35]. After targeted sequencing analysis on the validation cohort, an additional number of biomarkers could be detected for each amplicon. A differential analysis of all detected RNA editing sites or isoforms was performed on a large cohort of 410 participants for each amplicon analyzed and a “Target Editing Index” (TEI) index was carried out from this analysis. TEI index of the 8 selected targeted genes shows a very significant (adjusted *p* value ≤ 0.05) discrimination between patients suffering from depression (*n* = 267) and healthy controls (*n* = 143) (Fig. 4A). In addition, all significant biomarkers were combined with each other using a random forest machine learning approach leading to an AUC of 0.930 (CI 95% [0.879–0.982]), with a sensitivity of 84.0% and a specificity of 87.1%, allowing a clear separation of depressed patients from controls (Fig. 4B).

In order to validate the differential diagnostic performance of the 8 targeted genes panel between unipolar and BD patients, we measured RNA editing rate using ultra-deep targeted sequencing in a subgroup of 255 participants, coming from the discovery cohort: 160 unipolar and 95 bipolar depressed patients. For each amplicon analyzed, a differential analysis of all RNA editing sites/isoforms detected was carried out, leading to significant TEI index (adjusted *p* value ≤ 0.05) for 6 targeted genes (GAB2, IFNAR1, KCNJ15, LYN, MDM2 and PRKCB) (Fig. 4C). Then, all significant biomarkers were combined with each other using a random forest machine learning approach, leading to an AUC ROC curve of 0.935 (CI 95%: [0.882–0.988]), with a sensitivity of 90.9% and a specificity of 84.6% (Fig. 4D), allowing a clear separation of unipolar from BD patients. Interestingly, the machine learning approach allowed us to reduce the list number of targeted genes to as few as 6 for the differential diagnostic of BD, as compared to the 8 targeted genes panel discriminating healthy controls versus depressed patients.

## Discussion

The aim of our study was to identify a panel of blood biomarkers involved in depression, and to investigate their discriminative power in the differential diagnosis of BD. Based on our previous results [32, 33, 35, 65], we used RNA-Seq method and editome analysis to investigate blood transcriptome-wide RNA editing modifications in a discovery cohort of controls and depressed patients [66]. The analysis of the samples showed that RNA edition is mainly found in untranslated regions, has a homogeneous repartition all over the genome, and that no significant differences in Alu editing index (AEI) between controls and depressed patients are found. Thus, we can conclude that in this study the identification of differential RNA editing sites in blood is target-specific rather than due to a global RNA editing modification.

Then, we measured RNA editing modifications of the 8 selected targeted genes on a large validation cohort of 410 participants by multiplexed targeted sequencing. We confirm specific RNA editing signatures in the blood of depressed patients that allowed a biological detection of depression with high performance. A random forest machine learning approach was applied to optimize the diagnostic potential and reduce the number of genes from our panel, leading to a 6 targeted genes panel allowing differential diagnostic of BD with high specificity and selectivity.

The understanding of the pathophysiology of depression has progressed and several mechanisms have been involved, mainly the monoamine hypothesis, hypothalamic-pituitary-adrenal axis changes, neuroplasticity and neurogenesis, epigenetics and inflammation [8]. Alterations of peripheral inflammatory markers have consistently been involved in mood disorders, including unipolar depression and BD [67]. Previous studies on high-sensitivity C reactive protein (hs-CRP) have produced mixed results regarding the association between depression and hs-CRP [68]. In the present study, we did not find any significant modification in hs-CRP levels in unipolar or bipolar depressed patients. Elevated levels of peripheral proinflammatory mediators have been reported in BD, as well as in other mood disorders, and people with systemic autoimmune diseases have an increased risk of developing depression and BD [69]. More recently, it was shown that a combination of cytokines could correctly classify BD and MDD patients with 98.1% accuracy [70]. A wealth of studies have linked interferons to inflammation-induced changes in brain function and depression [71, 72], and our results are in phase with this litterature. RNA editing is strongly linked to interferon response [73], and polymorphisms in the promoter region of IFNAR1 can influence the risk of developing depression [74, 75]. Lyn plays an important role in the regulation of innate and adaptive immune responses, especially the inflammatory response to bacterial lipopolysaccharide [76]. Activation of Lyn directly enhances glutamatergic synaptic transmission and activates the mitogen-activated protein kinase (MAPK) signaling pathway, which subsequently increases the expression of Brain-Derived Neurotrophic Factor (BDNF) [77]. CAMK1D was shown as a key modulator of immune resistance [78]. Furthermore, single nucleotide polymorphisms (SNPs) of CAMK1D were associated with depressive episodes and suicide attempt status of depressed patients [79]. GAB2 might be an important regulator of the human Th2 immune response GAB2 [80]. Its overexpression makes neurons vulnerable, increasing tau phosphorylation, leading to Alzheimer’s Disease (AD) phenotype [81]. GAB2 genetic variation modulates AD risk via the alteration of both Aβ and tau pathology [82, 83]. Likewise, KCNJ15 in the Chinese population contributes to AD risk. These variants may exert their functional effects through the immune system [84]. The PRKCB gene expression in peripheral blood mononuclear cells (PBMC) was shown to be down-regulated in depressed patients [85], and an association with a combination of three SNPs at PRKCB gene with major depression was reported [86]. PRKCB also act as a regulator of the HPA axis response to stress, phosphorylating CREB1, which directly regulates the expression of BDNF, TrkB and the glucocorticoid receptor [20]. MDM2 catalyzes ubiquitination of β-arrestin, which is a target for antidepressants. In the CNS, MDM2 is involved in AMPAR surface expression during synaptic plasticity [87]. Thus, all the identified genes have a close relationship with neuronal and/or inflammatory mechanisms. The link between the observed RNA modifications and eventually their consequences in protein synthesis or stability remains to be demonstrated. Interestingly, preliminary data show that some of our biomarkers are modified in SH-SY5Y cells upon IFN activation (not shown). Further analyses should be performed to study the impact of editing variations at the protein level.

All patients were treated by different classes of medication, whose distribution was significantly different between the groups of patients. However, all the results were corrected by treatment to prevent a possible bias. Although this study is to our knowledge one of the largest studies on differential diagnostic of BD, future studies are needed to replicate our results in larger, unmedicated patient cohorts. Besides, the authors’ will is to expand the study of the panel biomarkers in other cohorts from other hospital centers.

In summary, through a transcriptome-wide RNA-Seq study in a discovery cohort that includes healthy volunteers and depressed patients, we have identified in blood RNA editing modifications in genes involved in different pathways relevant to mood disorders. We have confirmed this panel of biomarkers in a large validation cohort of depressed patients by ultra-deep targeted sequencing. In a second step, we have shown that a combination of 6 blood RNA editing-related biomarkers allows discriminating unipolar and BD, what may be crucial to improve BD diagnosis and orientate the treatment of millions of patients suffering from misdiagnosis. Our findings will contribute to a better understanding of the molecular pathophysiology of BD, and pave the way for the development of a diagnostic assay for BD with clinical application. This will change the game for the management of patients.

## Supplementary information

Suppl information

Suppl figure 1

Suppl figure 2

Suppl figure 3

Suppl figure 4

Suppl figure 5

Suppl Table 1

Suppl Table 2

Suppl Table 3

Suppl Table 4

Suppl Table 5
