# Supplementary material for: A game changer for bipolar disorder diagnosis using RNA editing-based biomarkers
Source: Transl Psychiatry. 2022 May 4;12:182. doi: 10.1038/s41398-022-01938-6 (PMC9064541; doi:10.1038/s41398-022-01938-6)
Supplement: Supplementary file 1 — Suppl information [file 41398_2022_1938_MOESM1_ESM.docx]

**Supplementary Figure Legends:**

Supplementary Table 1: Demographic and clinical characteristics of the study population included in the RNA-Seq study. Data are presented as the mean ± SEM; p-values of main characteristics are obtained with the Student’s t-test. MADRS: Montgomery-Åsberg depression rating scale; IDS-C30: The 30 item Inventory of Depressive Symptomatology; BMI: Body Mass Index.

Supplementary Figure 1: Overview of the Editome analysis pipeline. A: overview of workflow for RNA-Seq and editome analysis; B: overview of bioinformatics workflow for Targeted Next Generation Sequencing

Supplementary Figure 2: Correlation between clinical MADRS and IDS-C30 scores obtained for the depressed patients included in the discovery (A) and validation (B) cohorts. Graph shows the 95% Intervals confidence. The Pearson correlation coefficient and p-value are indicated.

Supplementary Figure 3: RNA editing site selection procedures from the 366 genes differentially edited between depressed patients (n=26) and controls (=31).

Supplementary Table 2: Functional categorization of the 366 genes differentially edited between depressed patients (n=26) and controls (n=31) based on gene ontology (GO) annotations. Shown are the results of top 20 GO over-representation tests for enrichment of Biological Process GO terms with differentially edited genes. The input into the GO enrichment analysis tools was the list of 366 genes differentially edited between depressed patients and controls. The p-values corrected using the Benjamini-Hochberg method for multiple testing are presented. FDR, False Discovery Rate; GO, Gene Ontology.

Supplementary Table 3: Reactome pathway enrichment analysis of the 366 genes differentially edited between depressed patients (n=26) and controls (n=31) in the discovery cohort. The table shows the significant pathways obtained performing over-representation analysis in Reactome. The ratio of the number of genes/total number of genes included in the pathway and p-value corrected using the Benjamini-Hochberg method for multiple testing are shown.

Supplementary Figure 4: Graphical representation of the top 20 GO term enrichment analysis for the 366 genes differentially edited between depressed patients (n=26) and controls (n=31) in the discovery cohort.

Supplementary Table 4: Reactome pathway enrichment analysis of the 7 identified genes analyzed in the discovery cohort.

Supplementary Table 5: List of 7 identified genes annotated through gene-disease association analysis.

Shown are the gene name, Ensembl ID, Uniprot ID, the full name of biomarkers, diseases associated to genes panel, categories of diseases associated to genes panel. All genes have been analyzed and their relationships with mental disorders, behavior and behavior mechanisms, nervous system diseases and immune system diseases MeSH categories have been calculated with DisGeNET database.

Supplementary Figure 5: Network of diseases associated with the 7 identified genes annotated by the DisGeNET database.
