# Supplementary figures and images for: A game changer for bipolar disorder diagnosis using RNA editing-based biomarkers
Source: Transl Psychiatry. 2022 May 4;12:182. doi: 10.1038/s41398-022-01938-6 (PMC9064541; doi:10.1038/s41398-022-01938-6)

Suppl Figure 1: Overview of the editome analysis pipeline

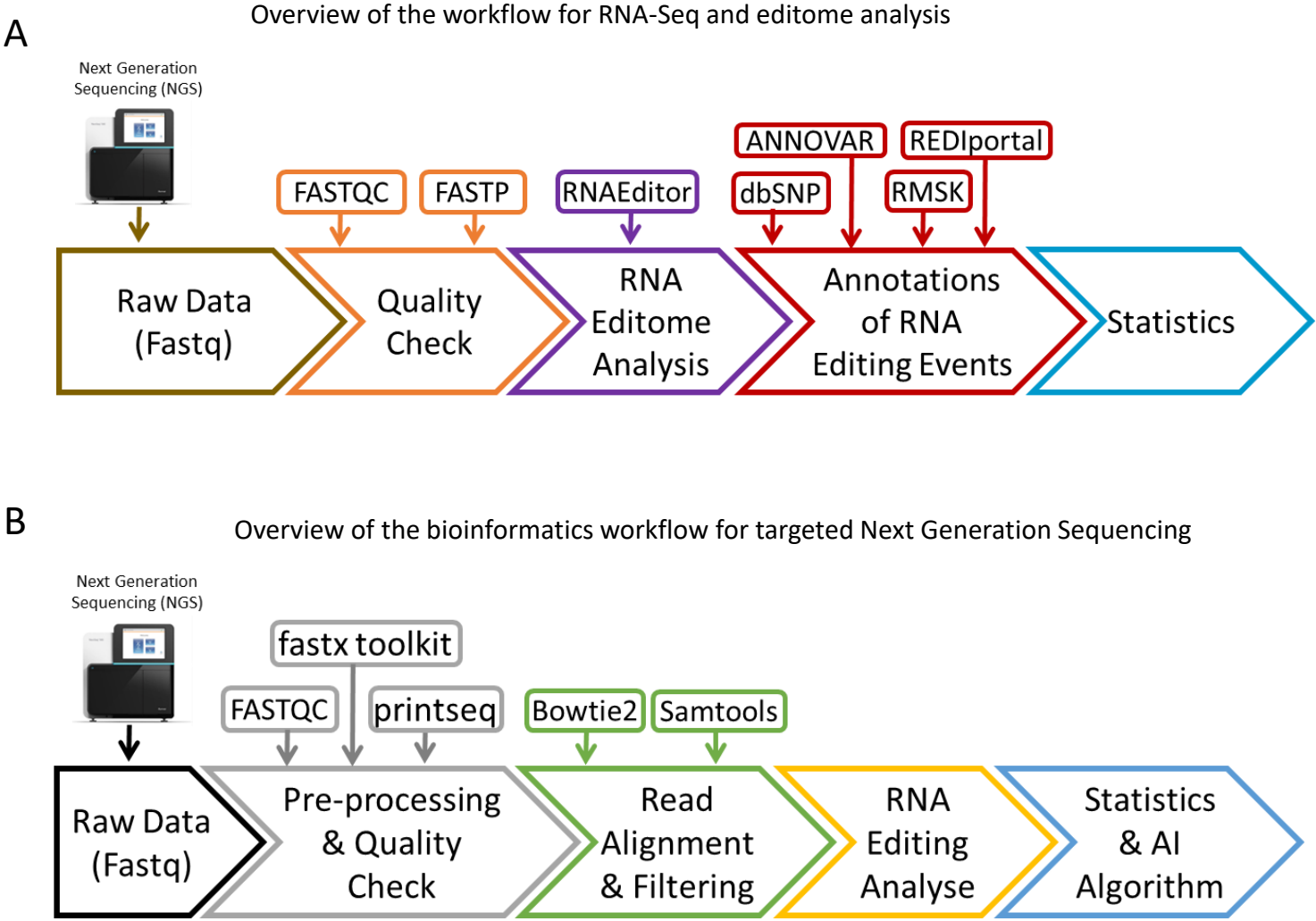

Supplement: Supplementary file 2 — Suppl figure 1 [file 41398_2022_1938_MOESM2_ESM.pdf]
