# Supplementary material for: A game changer for bipolar disorder diagnosis using RNA editing-based biomarkers
Source: Transl Psychiatry. 2022 May 4;12:182. doi: 10.1038/s41398-022-01938-6 (PMC9064541; doi:10.1038/s41398-022-01938-6)

Suppl Figure 2:

Correlation between clinical MADRS and IDS-C30 scores for the depressed patients included in the discovery (A) and validation (B) cohorts

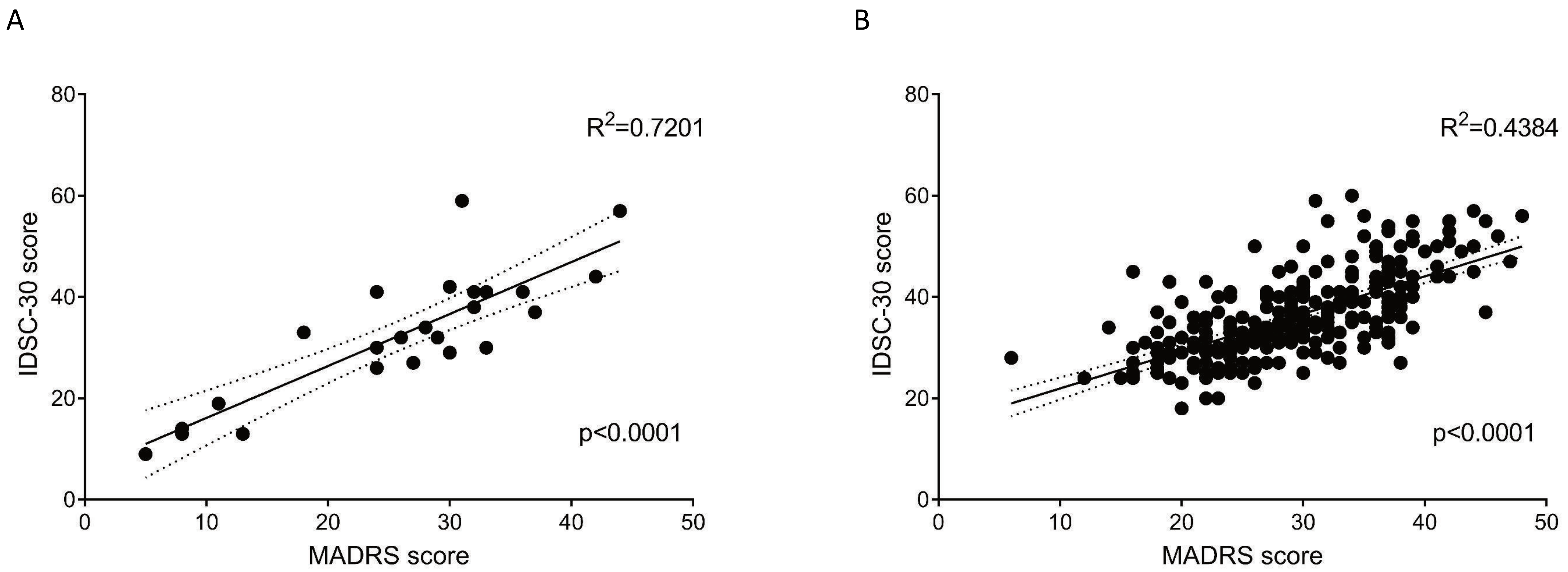

Supplement: Supplementary file 3 — Suppl figure 2 [file 41398_2022_1938_MOESM3_ESM.pdf]
