# Supplementary material for: A game changer for bipolar disorder diagnosis using RNA editing-based biomarkers
Source: Transl Psychiatry. 2022 May 4;12:182. doi: 10.1038/s41398-022-01938-6 (PMC9064541; doi:10.1038/s41398-022-01938-6)

Suppl Figure 4: Graphical representation of the top 20 GO term enrichment analysis for the 366 genes differentially edited between depressed patients (n=26) and controls (n=31)

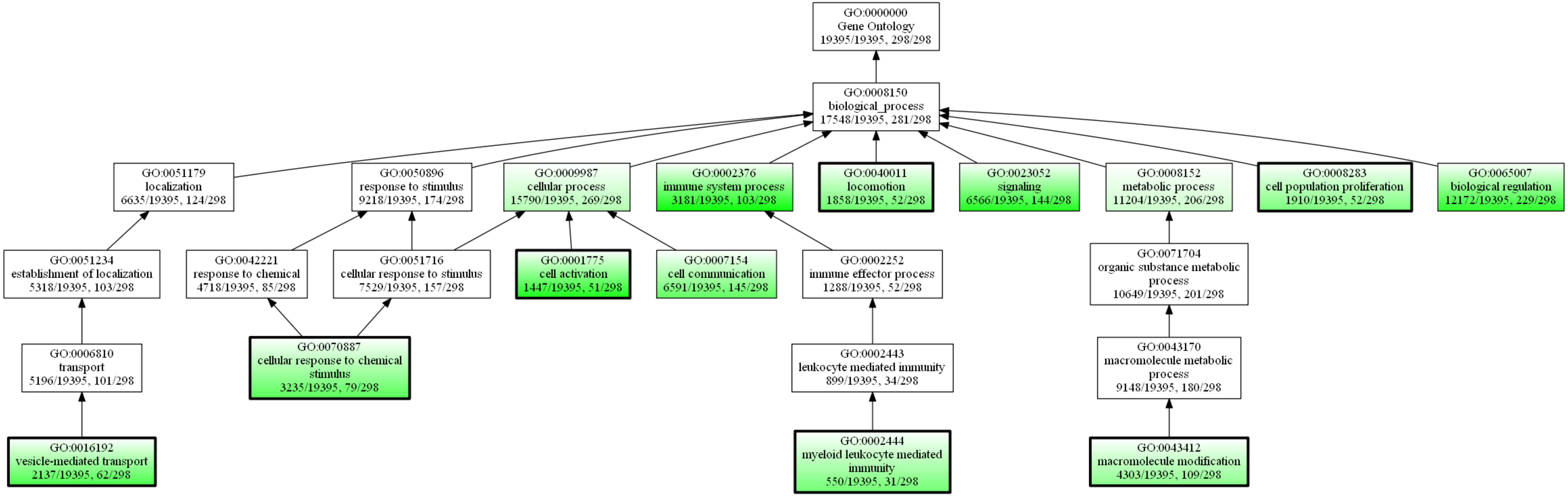

Supplement: Supplementary file 5 — Suppl figure 4 [file 41398_2022_1938_MOESM5_ESM.pdf]
