# Supplementary material for: A game changer for bipolar disorder diagnosis using RNA editing-based biomarkers
Source: Transl Psychiatry. 2022 May 4;12:182. doi: 10.1038/s41398-022-01938-6 (PMC9064541; doi:10.1038/s41398-022-01938-6)

Suppl figure 5: Network of diseases associated with the 7 identified genes annotated by the DisGeNET database

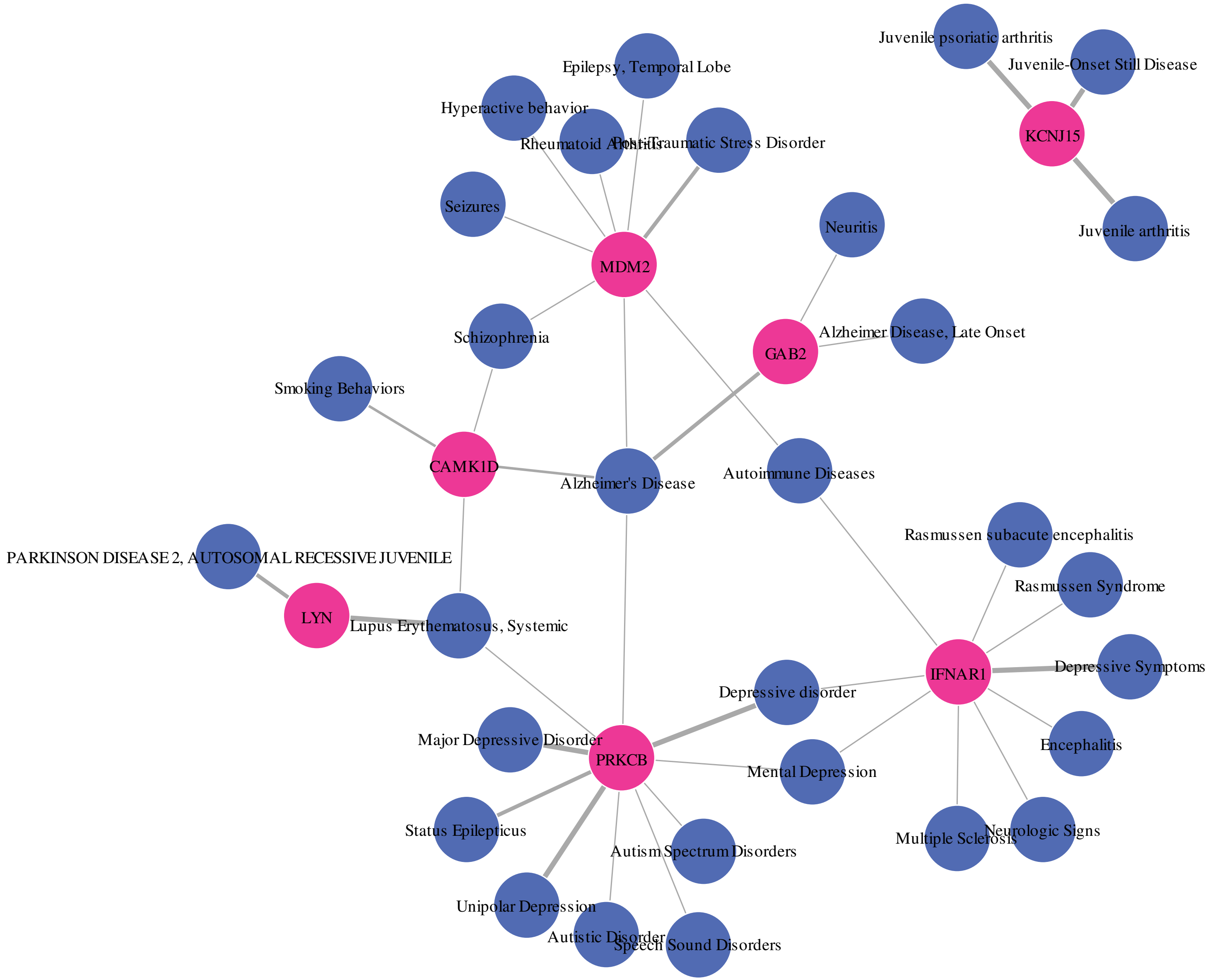

Supplement: Supplementary file 6 — Suppl figure 5 [file 41398_2022_1938_MOESM6_ESM.pdf]
