# Supplementary material for: A game changer for bipolar disorder diagnosis using RNA editing-based biomarkers
Source: Transl Psychiatry. 2022 May 4;12:182. doi: 10.1038/s41398-022-01938-6 (PMC9064541; doi:10.1038/s41398-022-01938-6)
Supplement: Supplementary file 7 — Suppl Table 1 [file 41398_2022_1938_MOESM7_ESM.pdf]

Suppl Table 1: Demographic and clinical characteristics of the study population included in the RNA-Seq study.

	Total Sample	Controls	Depressed	*P* Value
**Demographics**				
No. (%)	57 (100)	31 (54)	26 (46)	
Age (min-max), y	24-62	24-59	27-62	
Age, mean (SD), y	41.2 (9.7)	41.8 (9.8)	40 (9.7)	0.6
**Gender**				
Male, No. (%)	30 (53)	16 (52)	14 (54)	
Female, No. (%)	27 (47)	15 (48)	12 (46)	
**Clinical characteristics**				
MADRS score, mean (SEM)		0.8 (1.4)	26.0 (4.7)	<0.00001
IDSC30 score, mean (SEM)		2.7 (0.5)	32.6 (5.9)	<0.00001
BMI, mean, kg/m^2		25.3	23.3	0.08
