# Supplementary material for: A game changer for bipolar disorder diagnosis using RNA editing-based biomarkers
Source: Transl Psychiatry. 2022 May 4;12:182. doi: 10.1038/s41398-022-01938-6 (PMC9064541; doi:10.1038/s41398-022-01938-6)
Supplement: Supplementary file 8 — Suppl Table 2 [file 41398_2022_1938_MOESM8_ESM.pdf]

Suppl Table 2: Functional categorization of the 366 genes differentially edited between depressed patients (n=26) and controls (n=31) based on gene ontology (GO) annotations

Category	GO ID	GO term	p(FDR)
Biological Process	GO:0002682	regulation of immune system process	2.37E-12
Biological Process	GO:0050776	regulation of immune response	5.37E-10
Biological Process	GO:0002376	immune system process	1.09E-09
Biological Process	GO:0050778	positive regulation of immune response	7.68E-09
Biological Process	GO:0002768	immune response-regulating cell surface receptor signaling pathway	1.62E-08
Biological Process	GO:0002757	immune response-activating signal transduction	3.53E-08
Biological Process	GO:0002252	immune effector process	3.83E-08
Biological Process	GO:0002253	activation of immune response	3.83E-08
Biological Process	GO:0002429	immune response-activating cell surface receptor signaling pathway	3.83E-08
Biological Process	GO:0002684	positive regulation of immune system process	3.83E-08
Biological Process	GO:0002764	immune response-regulating signaling pathway	3.83E-08
Biological Process	GO:0045321	leukocyte activation	1.13E-07
Biological Process	GO:0001775	cell activation	1.67E-07
Biological Process	GO:0080090	regulation of primary metabolic process	1.67E-07
Biological Process	GO:0044093	positive regulation of molecular function	2.90E-07
Biological Process	GO:0006464	cellular protein modification process	3.36E-07
Biological Process	GO:0060255	regulation of macromolecule metabolic process	3.63E-07
Biological Process	GO:0048584	positive regulation of response to stimulus	3.89E-07
Biological Process	GO:0051171	regulation of nitrogen compound metabolic process	5.79E-07
Biological Process	GO:0006955	immune response	8.88E-07
