# Supplementary material for: A game changer for bipolar disorder diagnosis using RNA editing-based biomarkers
Source: Transl Psychiatry. 2022 May 4;12:182. doi: 10.1038/s41398-022-01938-6 (PMC9064541; doi:10.1038/s41398-022-01938-6)
Supplement: Supplementary file 9 — Suppl Table 3 [file 41398_2022_1938_MOESM9_ESM.pdf]

Suppl Table 3: Reactome pathway enrichment analysis of the 366 genes differentially edited between depressed patients (n=26) and controls (n=31)

Pathway ID	Pathway name	Event hierarchy	Entities ratio	p(FDR)
R-HSA-202427	Phosphorylation of CD3 and TCR zeta chains	Adaptative Immune System	16/45	8.99E-10
R-HSA-202433	Generation of second messenger molecules	Adaptative Immune System	16/58	1.79E-08
R-HSA-202430	Translocation of ZAP-70 to Immunological synapse	Adaptative Immune System	14/42	1.79E-08
R-HSA-389948	PD-1 signaling	Adaptative Immune System	14/45	3.27E-08
R-HSA-202403	TCR signaling	Adaptative Immune System	21/146	8.91E-07
R-HSA-388841	Costimulation by the CD28 family	Adaptative Immune System	19/97	1.30E-06
R-HSA-202424	Downstream TCR signaling	Adaptative Immune System	17/124	3.53E-05
R-HSA-877300	Interferon gamma signaling	Cytokine Signaling in Immune System	21/250	2.73E-03
R-HSA-2132295	MHC class II antigen presentation	Adaptative Immune System	15/148	4.53E-03
R-HSA-9616222	Transcriptional regulation of granulopoiesis	Developmental biology	9/71	2.72E-02
