# Supplementary material for: A game changer for bipolar disorder diagnosis using RNA editing-based biomarkers
Source: Transl Psychiatry. 2022 May 4;12:182. doi: 10.1038/s41398-022-01938-6 (PMC9064541; doi:10.1038/s41398-022-01938-6)
Supplement: Supplementary file 10 — Suppl Table 4 [file 41398_2022_1938_MOESM10_ESM.pdf]

Suppl Table 4: Reactome pathway enrichment analysis of the 7 identified genes analyzed in the discovery cohort

Pathway ID	Pathway name	Event hierarchy	p(FDR)
R-HSA-8939246	RUNX1 regulates transcription of genes involved in differentiation of myeloid cells	Gene Expression	3.95E-03
R-HSA-399719	Trafficking of AMPA receptors	Neuronal System	1.02E-02
R-HSA-399721	Glutamate binding, activation of AMPA receptors and synaptic plasticity	Neuronal System	1.02E-02
R-HSA-6804757	Regulation of TP53 Degradation	Gene Expression	1.02E-02
R-HSA-6806003	Regulation of TP53 Expression and Degradation	Gene Expression	1.02E-02
R-HSA-112314	Neurotransmitter receptors and postsynaptic signal transmission	Neuronal System	1.02E-02
R-HSA-512988	Interleukin-3, Interleukin-5 and GM-CSF signaling	Immune System	1.02E-02
R-HSA-1433557	Signaling by SCF-KIT	Signal Transduction	1.02E-02
R-HSA-112315	Transmission across Chemical Synapses	Neuronal System	2.56E-02
R-HSA-2730905	Role of LAT2/NTAL/LAB on calcium mobilization	Immune System	3.41E-02
R-HSA-112316	Neuronal System	Neuronal System	4.33E-02
R-HSA-9006934	Signaling by Receptor Tyrosine Kinases	Signal Transduction	4.33E-02
R-HSA-9645135	STAT5 Activation	Immune System	4.33E-02
R-HSA-9027283	Erythropoietin activates STAT5	Signal Transduction	4.33E-02
R-HSA-5633007	Regulation of TP53 Activity	Gene Expression	4.33E-02
R-HSA-114516	Disinhibition of SNARE formation	Hemostasis	4.33E-02
R-HSA-983705	Signaling by the B Cell Receptor (BCR)	Immune System	4.33E-02
R-HSA-9027277	Erythropoietin activates Phospholipase C Gamma (PLCG)	Signal Transduction	4.33E-02
R-HSA-210990	PECAM1 interactions	Hemostasis	4.33E-02
R-HSA-1640170	Cell Cycle	Cell Cycle	4.33E-02
