# Supplementary material for: A game changer for bipolar disorder diagnosis using RNA editing-based biomarkers
Source: Transl Psychiatry. 2022 May 4;12:182. doi: 10.1038/s41398-022-01938-6 (PMC9064541; doi:10.1038/s41398-022-01938-6)
Supplement: Supplementary file 11 — Suppl Table 5 [file 41398_2022_1938_MOESM11_ESM.pdf]

Suppl table 5: List of 7 identified genes annotated through gene-disease association analysis

Gene Name	Gene ID	Protein ID	Name	Related Disease Name	Related Disease Class
**CAMK1D**	ENSG00000183049	Q8IU85	calcium/calmodulin dependent protein kinase type 1D	Alzheimer's disease ; lupus erythematosus; schizophrenia; smoking behaviors	nervous system diseases; mental disorders; mental or behavioral dysfunction; immune system diseases
**GAB2**	ENSG00000033327	Q9UQC2	GRB2-associated-binding protein 2	Alzheimer's disease; Alzheimer disease, Late onset; neuritis;	nervous system diseases; mental disorders
**IFNAR1**	ENSG00000142166	P17181	interferon alpha and beta receptor subunt 1	mental depression; depressive disorder; depressive symptoms; drug-induced depressive state; Encephalitis; Rasmussen subacute encephalitis; Rasmussen syndrome; neurologic signs; multiple sclerosis; autoimmine disease; asthma. total IgE. SPT	behavior and behavior mechanisms; nervous system diseases; mental disorders; immune system diseases
**KCNJ15**	ENSG00000157551	Q99712	ATP-sensitive inward rectifier potassium channel 15	Juvenile arthritis; Juvenile psoriatic arthritis; Juvenile-Onset Still Disease	immune system diseases
**LYN**	ENSG00000166501	P07948	LYN proto-oncogene, Src family tyrosine kinase	lupus erythematosus, systemic; parkinson disease 2, autosomal recessive juvenile	nervous system diseases; mental disorders; behavior and behavior mechanisms; immune system diseases
**MDM2**	ENSG00000135679	Q00987	MDM2 proto-oncogene; E3 ubiquitin-protein ligase Mdm2	arthritis rheumatoid; autoimmune diseases; Alzheimer's disease; hyperactive behavior; epilepsy, temporal lobe; seizures; Post-Traumatic Stress Disorder; schizophrenia	nervous system diseases; mental disorders; behavior and behavior mechanisms; immune system diseases
**PRKCB**	ENSG00000166501	P05771	protein kinase C beta type	autistic disorder; autism spectrum disorders; major depressive disorder; depressive disorder; unipolar depression; mental depression; status epilepticus; lupus erythematosus, systemic; Alzheimer's disease; speech sound disorders	nervous system diseases; mental disorders; behavior and behavior mechanisms; immune system diseases
